# Risk factors for neurocognitive impairment in patients with benign intracranial lesions

**DOI:** 10.1038/s41598-019-44466-y

**Published:** 2019-06-10

**Authors:** Stefanie Bette, Julia M. Ruhland, Benedikt Wiestler, Melanie Barz, Bernhard Meyer, Claus Zimmer, Yu-Mi Ryang, Florian Ringel, Jens Gempt

**Affiliations:** 1 Department of Neuroradiology, Klinikum rechts der Isar, Technische Universität München, Munich, Germany; 2 Department of Neurosurgery, Klinikum rechts der Isar, Technische Universität München, Munich, Germany; 3 Department of Neurosurgery, University Medical Centre, Johannes-Gutenberg-University Mainz, Mainz, Germany; 4 Department of Diagnostic and Interventional Radiology and Neuroradiology, Universitätsklinikum Augsburg, Augsburg, Germany

**Keywords:** Neurology, Oncology

## Abstract

This study was designed to assess risk factors for neurocognitive impairment in patients with benign intracranial lesions including tumors and vascular lesions. 74 patients (29 m, 51 f, mean age 54.4 years) with surgery for benign intracranial lesions were included in this prospective single-center study. Extensive neuropsychological testing was performed preoperatively, including tests for *attention, memory* and *executive functions*. Furthermore, headache and depression were assessed using the german version of the HDI (IBK) and the BDI-II. Multiple linear regression analyses of the percentile ranks (adjusted for age, sex and education) including the parameters age, Karnofsky Performance Status Scale (KPS), mood, pain and lesion size were performed to identify risk factors for cognitive impairment. Using the Mann-Whitney U test, the influence of hemisphere and type of lesion (tumor/vascular) was assessed. Posthoc Bonferroni correction was performed. Poorer neurocognitive functions were observed only in the category *attention* in patients with higher age (divided attention, WMS) and reduced KPS (WMS). Lesion volume, mood, pain, hemisphere or the type of the lesion (tumor, vascular) were not identified as risk factors for poorer neurocognitive functions in patients with benign intracranial lesions. Age and KPS are the main risk factors for poorer neurocognitive functions in the category *attention* in patients with benign intracranial lesions. Knowledge of these risk factors might be important to find appropriate therapy regimes to improve cognitive functions and quality of life.

## Introduction

For patients with intracranial lesions, the most important recorded parameters are age, neurological status and functional independence, as measured by the Karnofsky Performance Status Scale (KPS)^1^. Recent studies analyzed the role of neurocognitive impairment for glioma patients and showed that cognitive function is a predictor for survival^2–5^. The most common test to evaluate cognitive function is the mini-mental state examination (MMSE)^6^. Extensive neurocognitive testing is time-consuming and therefore not routinely used. Tumor location and size, age, KPS and tumor grade as risk factors for neurocognitive impairment of glioma patients were identified^3,7^. Most studies about neurocognitive functions in brain tumor patients focus on fast growing tumors such as gliomas and/or metastases^3,7–12^. Benign lesions grow slowly, and therefore, less cognitive impairment was shown due to the plasticity of the brain^13–15^. Few studies have reported neurocognitive functions in meningioma patients or in patients with incidental meningiomas^16,17^; most of them, however, have focused on pre- and postoperative comparisons^14,18–23^. Risk factors for neurocognitive impairment, such as tumor size and location, were identified^20^. A few studies also analyzed cognitive functions in patients with pituitary adenomas and unruptured intracranial aneurysms^24–26^. Patients with pituitary adenomas with suprasellar extension showed preoperative cognitive dysfunction that resolved two months after surgery^25^. For patients with unruptered aneurysms a slight cognitive dysfunction was observed after surgery^26^.

Very little is known about neurocognitive functions in patients with intracranial vascular lesions (except for intracranial aneurysms^26,27^) or rare intracranial tumors. Furthermore, many previous studies showed the significant impact of pain on patients’ cognitive functions^28^. However, only a few studies assessed the relationship between trigeminal neuralgia and cognitive impairment^29,30^.

There are—to our knowledge—no studies about neurocognitive functions of patients with benign intracranial lesions that include benign tumors and vascular lesions in one cohort.

The aim of this study was to assess preoperative neurocognitive functions in patients with benign intracranial lesions, to identify risk factors for cognitive impairment.

## Methods

This prospective non-randomized single-center study was approved by the local ethics committee (Clinical Trial Registration Number: 3094/11) and conducted in accordance with the ethical standards of the 1964 Declaration of Helsinki and its later amendments^31^. Written informed consent was obtained from all study participants.

### Patient population

From September 2012 to December 2014, patients with surgery for a benign intracranial process were enrolled.

Inclusion criteria comprised informed consent, age ≥ 18 years, surgery for a benign intracranial process, preoperative magnetic resonance imaging (MRI) and sufficient knowledge of the German language. Exclusion criteria were: age < 18 years, pregnancy, missing or retrieved informed consent, missing surgery or malign intracranial processes. As the extended test battery comprises many partially complex tests, only patients with a preoperative mini-mental status examination (MMSE) ≥ 18 were included in the study.

### Study design

Preoperative tests were performed after informed consent and detailed information of the patient. Patients who met the inclusion criteria performed the basic test battery and the extended test battery as described below before surgery.

Age, preoperative KPS (ordinal scale 0–100 [%]) and tumor location (lobe, hemisphere) were recorded by qualified neurosurgeons for all patients.

### Basic test battery

This test battery includes the well-known MMSE^6^, which measures the patients’ basic cognitive functions.

### Extended test battery

Raw scores were adjusted for age, sex and education to a normative population (percentile ranks). Analyses were performed for the percentile ranks. For a few subtests, no percentile ranks were available: visual field subtests and Stroop’s failure. These data are not analyzed.

- Attention

This study uses the test battery of attentional performance (TAP), a computer-based test battery described by Zimmermann *et al*.^32^. This battery was used to assess a broad range of cognitive deficits in the category *attention* and was selected by a neuropsychologist. No standardized test protocol is known for the measurement of impairment of *attention* in brain tumor patients, the TAP test battery was used in a previous study about gliomas^3^.

### Alertness

The subtest *Alertness* examines the reaction time. Visual stimuli are provided either with (TAP alertness W_sound) or without an acoustic notification (TAP alertness W_O_sound). Data are shown in milliseconds (ms) for delay of the patients’ reaction for tests with/without acoustic notification.

### Divided attention

This subtest analyzes the simultaneous reaction to visual and acoustic stimuli (divided attention visual/auditory). Patients are challenged to react to both, visual stimuli (moving crosses, visual task) and acoustic stimuli (two consecutive sounds, auditory task). Data are shown as delay (ms) and mistakes/omissions.

### Visual field

This subtest examines the patients’ visual field. The patients are requested to fix on one central point. Visual stimuli are provided; data are recorded for the right/left and central field of view and are shown as delay (ms) and omissions. As only raw data, and no percentile ranks were available for these subtests, no further analyses were performed.

### Trail-Making-Test A (TMT-A)

This is a well-known test for visual attention. Patients are asked to connect numbers (1–25) in the right order with a pencil. The test measures the time needed to correctly join all numbers^33^. Trail-Making-Tests assess the relations between speed and fluid intelligence, the TMT-A uses simple tasks^34^.

### Wechsler Memory Scale (WMS)

For this study, two subtests of the *Wechsler Memory Scale revised* was used^35^. The test consists of multiple subtests and analyzes verbal and non-verbal short-term memory. Patients are asked to perform tasks directly in the two subtests - the block-span and the digit-span subtest - as described previously^3^. Memory span (ms) and work memory (wm) are analyzed for verbal (v) and non-verbal (nv) short-term memory.

Both tests analyze patients’ immediate memory. As only subtests of short-term memory are used in this study, the results are presented in the *attention* category.

(b) Memory

### Verbal Learning and Memory Test (VLMT)

VLMT - the German version of the well-known Rey Auditory-Verbal Learning Test - analyzes patients’ episodic memory function and consists of a learning and interference list with fifteen words each^36^. The learning list is read out five times (Dg1–5), followed by the interference list. Patients are asked to recite the words from the learning list directly (Dg6) and after 30 minutes (Dg7).

### Rey Osterrieth complex figure test (ROCF)

Visual memory function is analyzed by this test^37^. A geometrical figure is shown to the patients, and then they are asked to draw the figure immediately (ROCF copy) and after 30 minutes (ROCF delay).

(c) Executive functions

### Trail-Making-Test B (TMT-B)

In this subtest, patients are asked to connect letters and numbers with a pencil in the appropriate order (1-A-2-B-3-C)^33^. In contrast to the TMT-A, the TMT-B assesses patients’ ability to switch between tasks and therefore measures also fluid abilities^34^.

### Regensburg Word Fluency Test (RWT)

This test examines lexical and semantic fluency^38^. Patients are asked to say words with a specific letter (e.g., apple, auto, …) for one minute (lexical fluency). Semantic fluency is analyzed by naming words within a specific category (e.g., food). Furthermore, the ability to change between specific letters (turning lexical) and between specific categories (turning semantic) is tested.

### Stroop Word Color Test

This test is also known as “color-word-interference-test” and examines selective attention and patients’ ability to inhibit cognitive interference. First, patients read the names of different colors (blue, green, yellow and red) written in black (word reading). Secondly, the patients are asked to name colored lines (line naming). Thirdly, there are differences between the written color and the color of the word (e.g., the word red is written in blue; interference).

### Assessment of mood and pain

As mood and pain are known to influence neurocognition^28,39^, further measurements and tests were performed. To assess patients’ mood, the well-known Beck-Depression-Inventary II (BDI-II) was used in 61/80 patients^40^. The BDI-II score ranges from 0–63 (higher scores stand for higher extent of depression), the median of the normative population is 7.4 (population of n = 582 depressive patients and n = 260 healthy controls, manual of Hautziger *et al*.^41^).

Headache was assessed using the IBK, the german version of the Headache Disability Inventory (HDI) in 49/80 patients^42^. Headache is divided into four scales: no headache, slight headache, moderate headache, severe headache.

### Volumetric measurement

Pre- and postoperative volumetric measurement of the intracranial lesion was performed by a neuroradiologist by semi-automatic manual segmentation (IPlannet Cranial 3.0, Fa. Brainlab, Munich, Germany). T1-weighted images after contrast agent were used for contrast-enhancing processes.

### Statistical analysis

Statistical analysis was performed using IBM SPSS Statistics version 23.0 and 25.0 (SPSS Inc., IBM Corp., Armonk, NY, USA). Data are either shown as mean/standard deviation (normally-distributed data) or as median/interquartile range (IR; non-normally-distributed data). A multiple linear regression model was used to analyze the influence of different metric parameters (age, preoperative KPS, preoperative contrast enhancing tumor volume, mood, pain) on neurocognitive functions (attention, memory and executive functions). The correlation matrix shows linear/non-linear relationships between dependent and independent variables. Only linear relationships were included for regression analyses. Correlations between independent variables were assessed by Spearman correlations. Independent variables did not show strong correlations (r < 0.8)^43^, therefore all three variables were assessed in the regression analysis (Table 1). Scatter plots for residuals of multiple linear regression analyses are recorded. Bonferroni correction was performed for the number of multiple linear regression analyses (n = 29) To assess the influence of nominal parameters (hemisphere, tumor vs. vascular) on neurocognitive functions, Mann-Whitney U tests with posthoc Bonferroni correction were performed. Analyses were performed for percentile ranks (values adjusted for age, education and sex). A *P*-value of <0.05 was defined as significant.

**Table 1:** Correlation matrix between independent variables.

	Age	KPS	Tumor volume	Mood	Pain
Age	r = 1.0	P = 0.061, r = −0.219	P = 0.248, r = 0.144	P = 0.043, r = −0.271	P = 0.075, r = −0.271
KPS	P = 0.061, r = −0.219	r = 1.0	P = 0.021, r = −0.284	P = 0.382, r = −0.119	P = 0.614, r = 0.078
Tumor volume	P = 0.248, r = 0.144	P = 0.021, r = −0.284	r = 1.0	P = 0.560, r = 0.085	P = 0.910, r = −0.019
Mood	P = 0.043, r = −0.271	P = 0.382, r = −0.119	P = 0.560, r = 0.085	r = 1.0	P = 0.040, r = 0.314
Pain	P = 0.075, r = −0.271	P = 0.614, r = 0.078	P = 0.910, r = −0.019	P = 0.040, r = 0.314	r = 1.0

### Ethical approval and informed consent

The study was conducted in accordance with the ethical standards of the 1964 Declaration of Helsinki and its later amendments and approved by the local ethics committee (Ethics committee technical university munich). Informed consent was signed by all study participants.

## Results

### Patient population

81 patients met initially the inclusion criteria and were included in the study. One patient did receive neuroradiological intervention for an intracranial aneurysm and was excluded from the study. 6 patients presented with trigeminal nerve neuralgia and were excluded due to a missing intracranial lesion. Therefore, the study population comprises 74 patients with the following benign lesions: meningioma (n = 31), pituitary adenoma (n = 14), vestibular schwannoma (n = 8), cavernoma (n = 6), intracranial aneurysm (n = 4), pineocytoma (n = 2), arterial–venous malformation (AVM; n = 2), hemangiopericytoma (n = 1), clivus chordoma (n = 1), colloidal cyst (n = 1), subependymoma (n = 1) and others (n = 3). For further analysis, lesions are assessed in two groups: tumor (including meningioma, pituitary adenoma, vestibular schwannoma, pineocytoma, hemangiopericytoma, clivus chordoma, colloidal cyst and subependymoma) and vascular syndrome (including cavernoma, AVM and aneurysm). Median preoperative KPS was 100% (range 40–100). The median preoperative (contrast enhancing) volume of the intracranial lesions was 2.5 cm³ (IR 0.8–10.4 cm³) (Table 2).

**Table 2:** Patient population.

Age (mean [range]) Sex, male	54.6 years [range 24.0–77.0years] 28/74
*Tumor histology*	
- meningioma	31/74
- pituitary adenoma	14/74
- vestibular schwannoma	8/74
- cavernoma	6/74
- intracranial aneurysm	4/74
- pineocytoma	2/74
- arterio-venous malformation	2/74
- hemangiopericytoma	1/74
- clivus chordoma	1/74
- colloidal cyst	1/74
- subependymoma	1/74
- others	3/74
*Main tumor location*	
- frontal lobe	28/74
- temporal lobe	9/74
- parietal lobe	3/74
- mid line	17/74
- infratentorial	16/74
- ventricle	1/74
*Hemisphere*	
- right	28/74
- left	24/74
- median	22/74
*Involvement of the skull base*	
- Anterior	20/74
- Middle	46/74
- Posterior	20/74
*Involvement of the insula*	2/74
*Intracerebral*	51/74
*Education*	
- elementary school	32/69
- intermediate education	20/69
- high school	17/69
Recurrent disease	14/74

### Basic test battery

#### MMSE

Median preoperative MMSE was 29.0 (IR 27.8–30.0). Patients’ age showed a significant negative correlation to MMSE (r = −0.303, P = 0.009). Volume of the intracranial lesion did not correlate to preoperative MMSE scores (r = 0.131, P = 0.296).

Furthermore, the type of intracranial lesion and location (lobe/hemisphere) were not significantly associated with MMSE.

### Extended test battery

#### Attention

**Figure 1:**
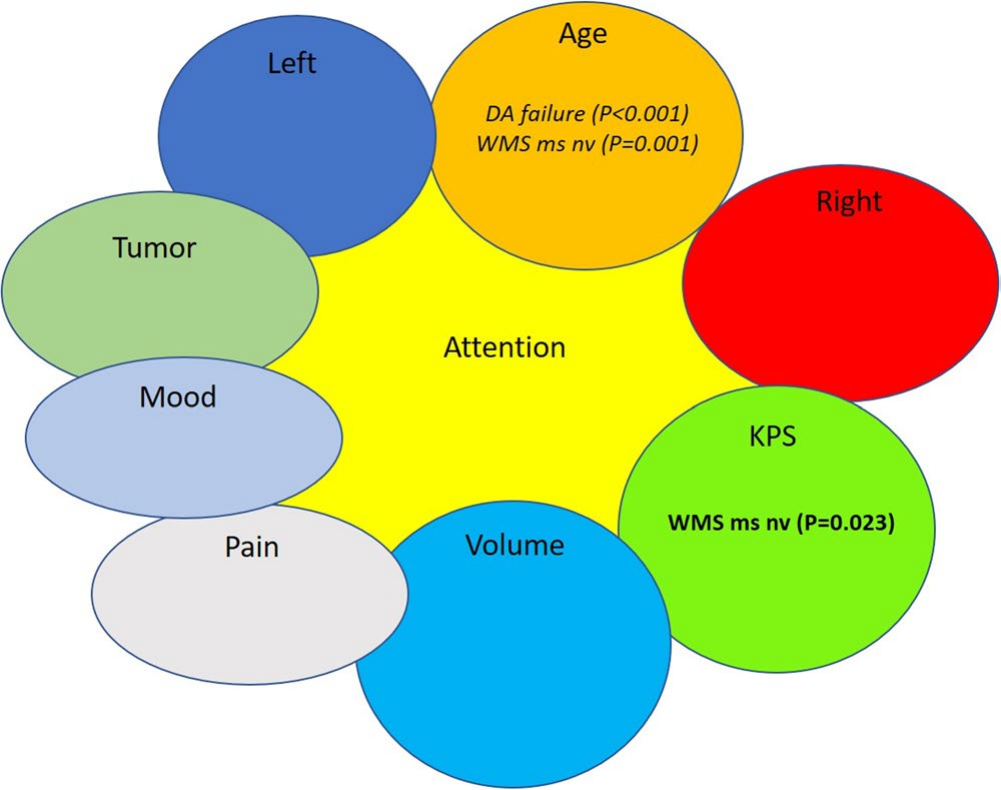
Risk factors for poorer cognitive functions in the *attention* category. Poorer cognitive functions are shown in *Italics*, and better neurocognitive functions is shown in Bold. DA: divided attention. WMS ms nv: Wechsler Memory Scale memory span non-verbal.

**Figure 2:**
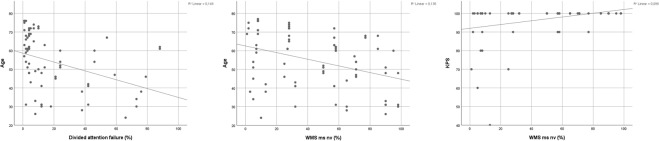
Scatter plots for risk factors age and KPS and neurocognitive functions in the subtests DA failure (divided attention failure) and WMS ms nv (Wechsler Memory Scale memory span non-verbal). KPS: Karnofsky Performance Status Scale.

In the analysis of the percentile ranks, higher age predicted poorer functions only in 2/12 subtests (divided attention failure, WMS ms nv), higher KPS predicted better functions in 1/12 subtests (WMS ms nv). Lesion volume, pain and mood did not show significant results in this analysis. (Figs 1 and 2, Table 3).

**Table 3:** Attention.

			Standardized R²	F	Model significance	Adjusted P-Value	Condition index	Durbin Watson	Independent variables				
									Age	KPS	Tumor volume	Mood	Pain
**Attention**	**TAP**	**Alertness W_O_sound**	0.037	1.815	0.154	1.0	24.370	2.362	n.s.	n.s.	n.s.	—	—
		**Alertness W_sound**	0.015	1.322	0.276	1.0	24.370	2.298	n.s.	n.s.	n.s.	—	—
		**Alertness phasic**	0.064	3.150	0.050		20.208	2.205	—	n.s.	n.s.	—	—
		**Divided attention visual**	0.032	2.028	0.140	1.0	20.205	2.088	—	n.s.	n.s.	—	—
		**Divided attention auditive**	0.006	1.056	0.394	1.0	21.844	2.147	—	n.s.	n.s.	n.s.	n.s.
		Divided attention failure*	0.209	6.468	0.001	***0.029***	24.241	1.650	<*0.001*	n.s.	n.s.	—	—
		**Divided attention selected**	0.107	2.110	0.102	1.0	10.039	1.991	n.s.	—	n.s.	n.s.	n.s.
	**TMT-A**	**TMT-A**	0.069	3.351	0.042		22.884	1.810	—	n.s.	n.s.	—	—
	**WMS**	**WMS ms v**	0.032	3.435	0.068		19.237	2.012	—	n.s.	—	—	—
		**WMS wm v**	0.022	1.722	0.187	1.0	7.874	1.923	n.s.	—	n.s.	—	—
		WMS ms nv*	0.226	7.320	<0.001	***0.029***	24.674	2.365	*0.001*	**0.023**	n.s.	—	—
		**WMS wm nv**	0.163	2.897	0.036	1.0	22.238	2.233	n.s.	n.s.	n.s.	—	n.s.
**Memory**	**VLMT**	**VLMT Dg1**	0.087	1.906	0.132	1.0	9.970	2.292	n.s.	—	n.s.	n.s.	n.s.
		**VLMT Dg5**	0.187	3.238	0.023	0.667	22.238	2.154	n.s.	n.s.	n.s.	—	n.s.
		**VLMT Dg1-5**	0.218	3.722	0.013	0.377	22.238	2.015	n.s.	n.s.	n.s.	—	n.s.
		**VLMT Dg6**	0.053	1.546	0.210	1.0	22.238	2.033	n.s.	n.s.	n.s.	—	n.s.
		**VLMT Dg7**	0.108	2.185	0.091	1.0	22.238	2.084	n.s.	n.s.	n.s.	—	n.s.
		**VLMT Dg5-6**	−0.005	0.942	0.431	1.0	3.619	2.413	—	—	n.s.	n.s.	n.s.
		**VLMT Dg5-7**	−0.015	0.806	0.499	1.0	8.809	2.120	n.s.	—	n.s.	—	n.s.
	**ROCF**	**ROCF copy**	0.123	3.955	0.027	0.783	8.376	1.949	n.s.	—	—	—	n.s.
		**ROCF delay**	0.075	2.710	0.079	1.0	8.376	2.461	n.s.	—	—	—	n.s.
**Executive functions**	**Stroop’s**	**Stroop’s word reading**	−0.009	0.859	0.469	1.0	9.202	1.934	n.s.	—	n.s.	n.s.	—
		**Stroop’s naming**	0.030	1.283	0.297	1.0	26.025	1.821	—	n.s.	n.s.	n.s.	n.s.
		**Stroop’s interference**	0.106	2.825	0.050	1.0	26.025	1.682	—	n.s.	n.s.	n.s.	—
	**RWT**	**RWT lexical**	−0.008	0.921	0.463	1.0	22.280	1.947	—	n.s.	n.s.	n.s.	n.s.
		**RWT semantic**	0.248	3.502	0.012	0.348	24.942	2.151	n.s.	n.s.	n.s.	n.s.	n.s.
		**RWT turning lexical**	0.139	2.577	0.054	1.0	22.238	1.317	n.s.	n.s.	n.s.	—	n.s.
		**RWT turning semantic**	0.230	4.884	0.006	0.174	19.155	1.843	—	n.s.	n.s.	—	n.s.
	**TMT-B**	**TMT-B**	0.096	1.761	0.150	1.0	28.975	1.524	n.s.	n.s.	n.s.	n.s.	n.s.

Multiple linear regression analysis for the *attention* category. Only significant p-values (<0.05) are shown. Better neurocognitive functions are listed in Bold, poorer neurocognitive functions in *Italic*.

#### Memory

In the category *memory* none of the variables (age, KPS, tumor volume, mood, pain) predicted better or poorer neurocognitive functions (Table 3).

#### Executive functions

In the category *executive functions*, also none of the parameters predicted better or poorer neurocognitive functions (Table 3).

#### Tumor location/type of lesion

Prior to these analyses, important patient variables (age, mood, pain, tumor volume) are shown and compared in the different groups (divided in tumor location/hemisphere, and type of the lesion [tumor, vascular lesion]) (Table 4). Patients with tumors were significantly older than patients with vascular lesions (median 56.4 y. vs. 45.9 y, P = 0.031, U = 225, Z = −2.157). Patients with tumors in the left hemisphere showed significantly higher scores of the BDI-II (10.0 vs. 6.0, P = 0.049, U = 252, Z = −1.966). Tumor volume was significantly lower for tumors affecting the left hemisphere (1.6 cm³ vs. 6.2 cm³, P = 0.024, U = 348, Z = −2.264).

**Table 4:** Comparison of variables age, mood, pain and tumor volume in the different groups.

Groups	Variables							
	Age	P-value	Mood	P-value	Pain	P-value	Volume	P-value
Right hemisphere affected	52.8 y.	0.093	8.5	0.704	3.0	0.475	2.7 cm³	0.738
Right hemisphere not affected	58.6 y.		5.0		0.0		1.7 cm³	
Left hemisphere affected	54.1 y.	0.604	10.0	*0.049*	4.0	0.084	1.6 cm³	*0.024*
Left hemisphere not affected	55.6 y.		6.0		0.0		6.2 cm³	
Tumor	56.4 y.	*0.031*	8.5	0.36	2.0	0.627	3.0 cm³	0.114
Vascular lesion	45.9 y.		2.0		0.0		1.0 cm³	

Median scores are shown. For mood, the scores of the BDI-II are reported (points), for pain, the scores of the IBK (points). Data of volume are shown as the volume of the contrast enhancing preoperative tumor (cm³); y = years.

Tests for independent samples showed no significant differences of neurocognitive functions in all three categories in patients with tumors or vascular lesions, lesions in the left or right hemisphere.

#### Mood and pain

The median preoperative BDI-II of the patient cohort was 8.0 (IR: 1.3–15.0), therefore slightly higher than the normal population (median 7.4).

Higher scores of the BDI-II predicted poorer cognitive functions in none of the subtests (Table 3).

17/44 patients did not report preoperative headache, 15/44 patients presented with slight, 7/44 patients with moderate and 5/44 patients with severe headache.

Also pain did not predict poorer or better neurocognitive functions in all three categories (Table 3).

## Discussion

This prospective single-center study analyzed neurocognitive functions in 74 patients with benign intracranial lesions including meningiomas, pituitary adenomas and vestibular schwannomas and vascular lesions (aneurysm, cavernoma). Higher age predicted poorer functions only in subtests of the category *attention* (divided attention, WMS), not in the categories *memory* and *executive functions*. Lower KPS predicted poorer functions only in one subtest (WMS) in the category *attention* (WMS). Higher lesion volume, pain, mood, hemisphere or the type of the lesion (tumor, vascular) did not predict poorer or better neurocognitive functions in any of the three categories.

Analyses were performed for percentile ranks (values adjusted for age, sex and education). Most studies about neurocognitive functions in patients with benign intracranial lesions are either performed with analyses of values adjusted for age, sex and education^22,46–48^ or with analyses of matched-paired or healthy controls^17,21,49^. This prospective study only includes patients and no healthy controls, therefore analyses of values adjusted for age, sex and education (percentile ranks) were performed.

The main risk factors for poorer neurocognitive functions identified in this study cohort were higher age and reduced KPS. These results are similar to those of previous studies that included brain tumor patients^3,8^. Underlining the results of a previous study, age and KPS are more important risk factors than tumor location and size; these results are also confirmed in the patient cohort with benign intracranial lesions^3^.

For divided attention, significant poorer functions for patients with higher age. Poorer functions in immediate memory (analyzed by subtests of the Wechsler memory scale [WMS]) were observed for patients with higher age and lower KPS. These findings are in common with previous studies as age is a known predictor for cognitive dysfunction^44,45^.

Pain, especially chronic pain, is a known risk factor for neurocognitive impairment in all three categories^28^. Previous studies reported impaired working memory and poorer scores in the Wechsler Memory Scale for patients with chronic and induced pain^50–52^. The exact pathomechanisms are still unknown; studies showed that patients with chronic pain show poorer memory function than patients with induced pain, suggesting also other factors than pain might be attributable for cognitive impairment^51^. In this patient cohort, pain was not shown as a significant predictor for poorer neurocognitive functions. This might be explained by the fact that patients with tumors or vascular lesions do not present with pain, but with other symptoms like seizures or neurological deficits.

Mood was not shown as significant predictor for cognitive dysfunction in this patient cohort. These results are contrary to previous findings that showed depression as an important predictor for neurocognitive impairment^53–56^. In the patient cohort of this study the values of the BDI-II were comparable to the values of the normal population. Most studies showed cognitive impairment mostly for patients with major depression, this might explain the conflicting results.

Episodic memory function (analyzed by the Verbal Learning and Memory Test [VLMT]) was poorer in patients with higher age and lower KPS. These results are in common with a previous study that showed poorer episodic memory function in a cognitively normal elderly cohort suggesting dysfunction in the posterior cingulate region as an important factor^57^.

Lesion volume was not shown as a significant predictor for poorer cognitive. Previous studies reported that verbal fluency was impaired in patients with larger tumor volume^3,20^. These discrepancies might be explained by the patient cohort of this study. We included only patients with benign, predominantly small lesions. Another explanation for these findings might be neuroplasticity.

Neuroplasticity is a known phenomenon for patients with infiltrating brain tumors, especially slowly growing tumors like diffuse gliomas^58^. In this cohort we assessed benign and therefore slowly growing lesions, therefore neuroplasticity might influence the results of this study. This might also explain the results that mainly age and KPS affect patients’ cognitive functions.

No significant differences between vascular and tumoral lesions were observed in this patient cohort. These findings might be explained by the fact that only benign tumors were included in this study.

Previous studies reported cognitive functions for patients with predominantly only one tumor/lesion entity, e.g. meningioma, pituitary adenoma, trigeminal neuralgia or aneurysm^16,19,24–27,30^. In this study, patients with different intracranial lesion were assessed in one cohort. This introduces a large heterogeneity of the cohort, and it is not possible to draw conclusions for one tumor entity. However, cognitive functions for single entities were analyzed in previous studies. This study points out that in a cohort of patients with tumoral and vascular lesions the type of the lesion is no predictor for neurocognitive dysfunction. The main predictors for cognitive dysfunction are the known variables – age and KPS.

This study has limitations. The main limitation is the high variety of diseases and therefore a low number, respectively. However, rare tumors such as chordoma and subependymoma as well as rare vascular lesions like cavernomas were assessed. No conclusions can be drawn for single lesion entities according to the results of this study.

No comparisons between the study population and a normative cohort were performed; this, however, has been shown in previous studies for patients with benign intracranial tumors^17,21,22,47,48,59^. The results of this study are mainly compared to studies on meningioma patients, also the cited studies with comparisons to the normative cohort were mainly performed on meningioma patients. However, the patient cohort of this study only includes 31/80 meningioma patients which might involve a bias. As described above, only a few studies previously assessed cognitive functions in patients with other benign intracranial tumors and vascular lesions, therefore comparability is low and further studies are necessary to draw conclusions and to assess cognitive functions in comparison to a normative cohort in patients with rare intracranial lesions.

Another limitation might be the manual segmentation of the lesions. Especially for small lesions and for lesions at the skull base this might introduce a bias due to over- or underestimation of lesion volume^60^.

## Conclusions

Main risk factors for poorer neurocognitive functions in the category *attention*, not in the categories *memory* and *executive functions*, are higher age and reduced KPS in patients with benign intracranial lesions. Higher lesion volume, lesion location, type of the lesion (tumor, vascular), mood and pain did not predict poorer or better neurocognitive functions. As neurocognitive impairment influences patients’ quality of life, knowledge of these risk factors is important to perform neuropsychological testing and, if necessary, engineer appropriate therapy regimes.

## Supplementary information

Supplementary Info
