# Supplementary material for: Risk factors for neurocognitive impairment in patients with benign intracranial lesions
Source: Sci Rep. 2019 Jun 10;9:8400. doi: 10.1038/s41598-019-44466-y (PMC6557851; doi:10.1038/s41598-019-44466-y)
Supplement: Supplementary file 1 — Supplementary Info [file 41598_2019_44466_MOESM1_ESM.pdf]

**Risk factors for neurocognitive impairment in patients with benign intracranial lesions**

Stefanie Bette, Julia M. Ruhland, Benedikt Wiestler, Melanie Barz, Bernhard Meyer, Claus Zimmer, Yu-Mi Ryang, Florian Ringel, Jens Gempt

**Supplemental Table 1 Correlation matrix between dependent and independent variables**

| | | Age | KPS | Tumor volume | Mood | Pain |
|---|---|---|---|---|---|---|
| **Attention** | **Alertness W_O_sound** | Yes | Yes | Yes | No | No |
| | **Alertness W_sound** | Yes | Yes | Yes | No | No |
| | **Alertness phasic** | No | Yes | Yes | No | No |
| | **Divided attention visual** | No | Yes | Yes | No | No |
| | **Divided attention auditive** | No | Yes | Yes | Yes | Yes |
| | **Divided attention failure** | Yes | Yes | Yes | No | No |
| | **Divided attention selected** | Yes | No | Yes | Yes | Yes |
| | **TMT-A** | No | Yes | Yes | No | No |
| | **WMS ms v** | No | Yes | No | No | No |
| | **WMS wm v** | Yes | No | Yes | No | No |
| | **WMS ms nv** | Yes | Yes | Yes | No | No |
| | **WMS wm nv** | Yes | Yes | Yes | No | Yes |
| **Memory** | **VLMT Dg1** | Yes | No | Yes | Yes | Yes |
| | **VLMT Dg5** | Yes | Yes | Yes | No | Yes |
| | **VLMT Dg1-5** | Yes | Yes | Yes | No | Yes |
| | **VLMT Dg6** | Yes | Yes | Yes | No | Yes |
| | **VLMT Dg7** | Yes | Yes | Yes | No | Yes |
| | **VLMT Dg5-6** | No | No | Yes | Yes | Yes |
| | **VLMT Dg5-7** | Yes | No | Yes | No | Yes |
| | **ROCF copy** | Yes | No | No | No | Yes |
| | **ROCF delay** | Yes | No | No | No | Yes |
| **Executive functions** | **Stroop's word reading** | Yes | No | Yes | Yes | No |
| | **Stroop's naming** | No | Yes | Yes | Yes | Yes |
| | **Stroop's interference** | No | Yes | Yes | Yes | No |
| | **RWT lexical** | No | Yes | Yes | Yes | Yes |
| | **RWT semantic** | Yes | Yes | Yes | Yes | Yes |
| | **RWT turning lexical** | Yes | Yes | Yes | No | Yes |
| | **RWT turning semantic** | No | Yes | Yes | No | Yes |
| | **TMT-B** | Yes | Yes | Yes | Yes | Yes |

Yes: Linear correlation between variables; No: non-linear correlation between variables.

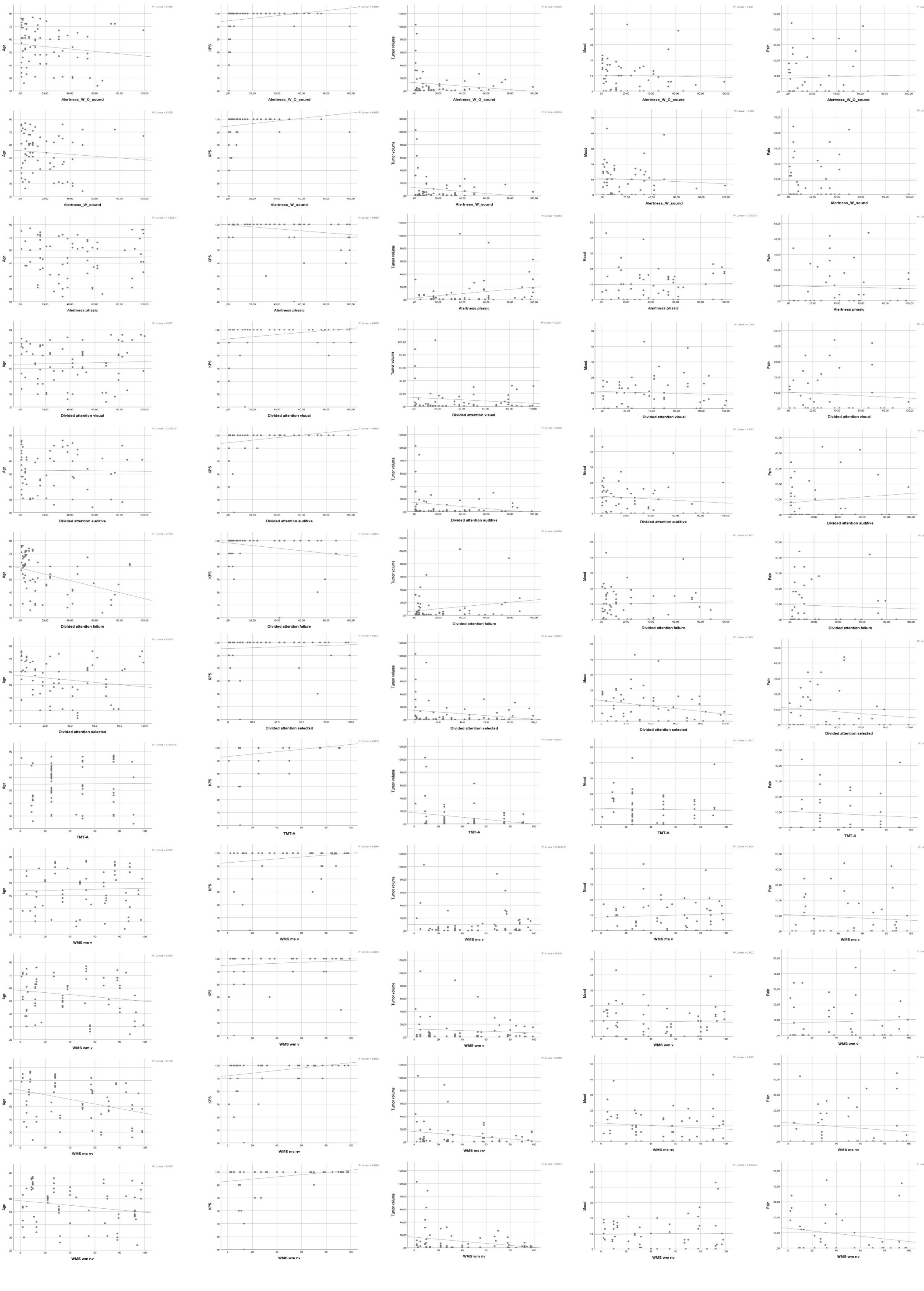

Age
KPS
Tumor volume
Mood
Pain
Alertness_W_O_sound
Alertness_W_sound
Alertness phasic
Divided attention visual
Divided attention auditive
Divided attention failure
Divided attention selected
TMT-A
WMS ms v
WMS wm v
WMS ms nv
WMS wm nv

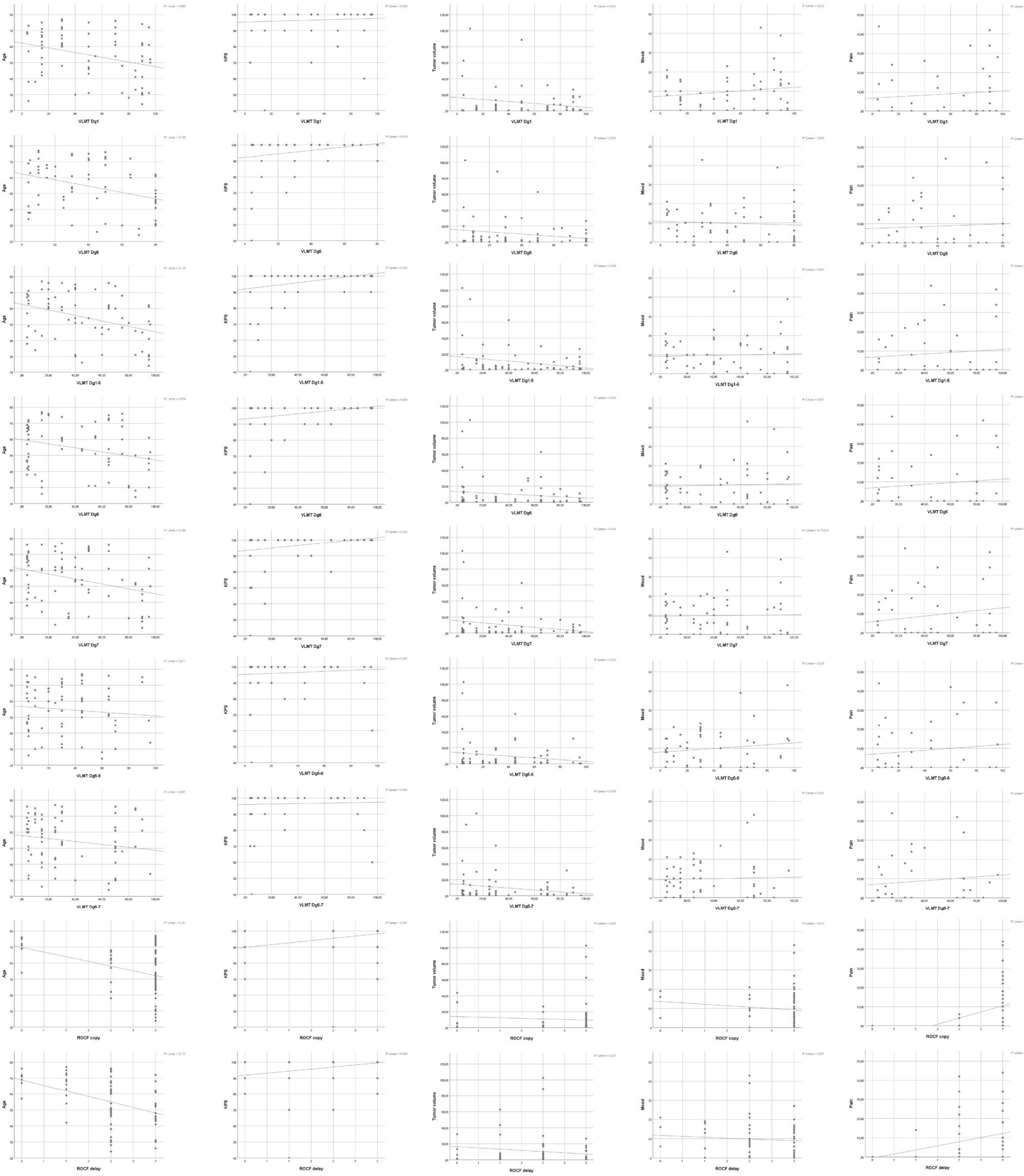
Age
KPS
Tumor volume
Mood
Pain
VLMT Dg1
VLMT Dg5
VLMT Dg1-5
VLMT Dg6
VLMT Dg7
VLMT Dg5-6
VLMT Dg5-7
ROCF copy
ROCF delay

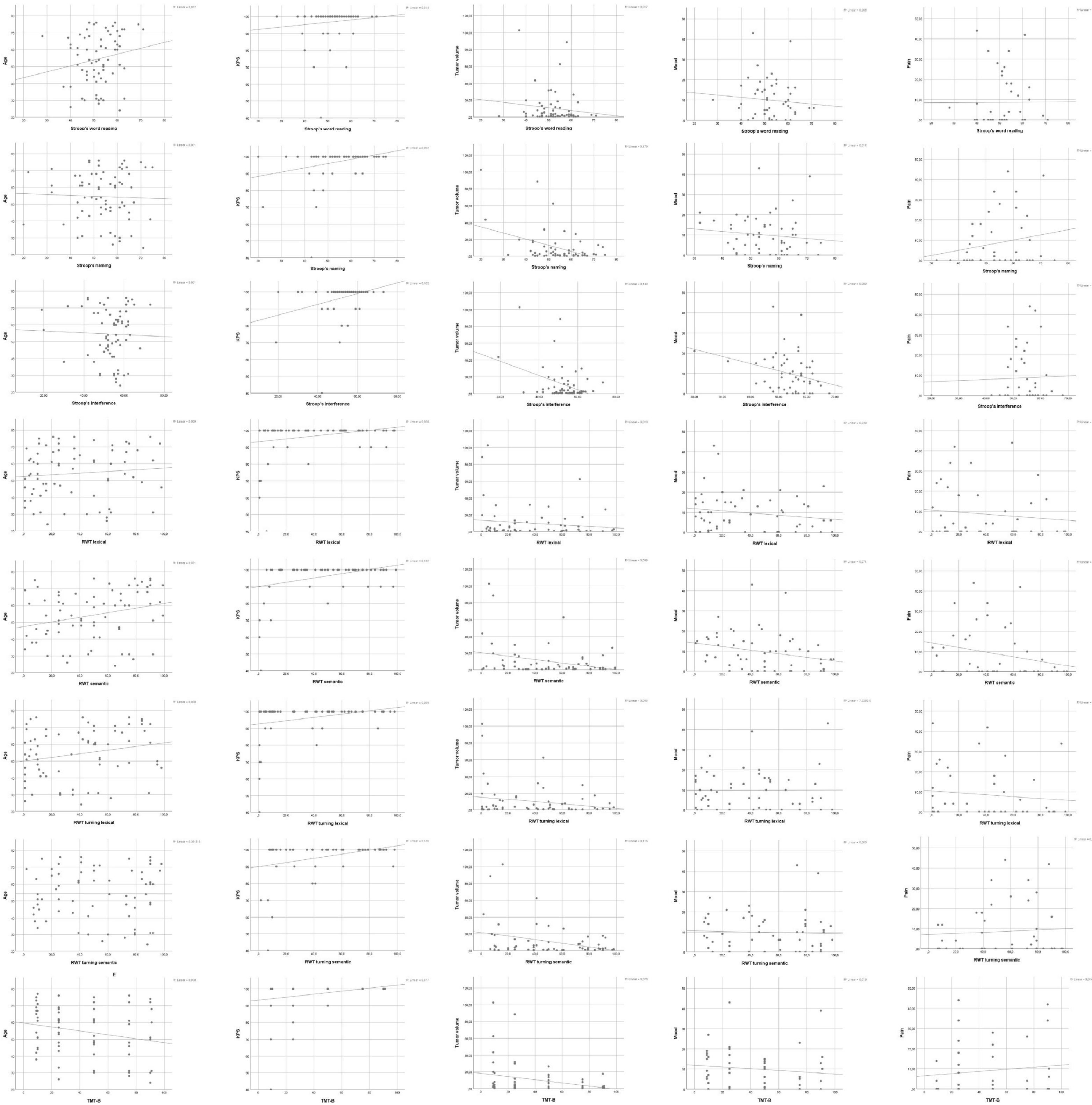

Age
KPS
Tumor volume
Mood
Pain
Stroop's word reading
Stroop's naming
Stroop's interference
RWT lexical
RWT semantic
RWT turning lexical
RWT turning semantic
E
TMT-B

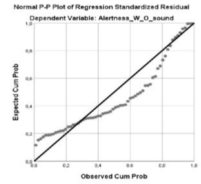
Normal P-P Plot of Regression Standardized Residual
Dependent Variable: Alertness_W_O_sound
Expected Cum Prob
Observed Cum Prob
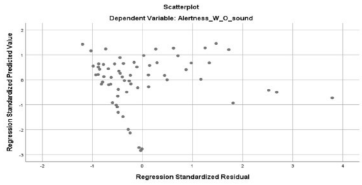
Scatterplot
Dependent Variable: Alertness_W_O_sound
Regression Standardized Predicted Value
Regression Standardized Residual
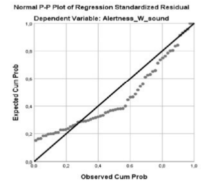
Normal P-P Plot of Regression Standardized Residual
Dependent Variable: Alertness_W_sound
Expected Cum Prob
Observed Cum Prob
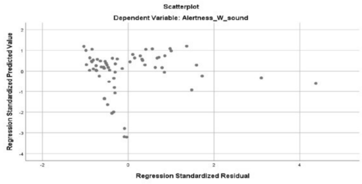
Scatterplot
Dependent Variable: Alertness_W_sound
Regression Standardized Predicted Value
Regression Standardized Residual
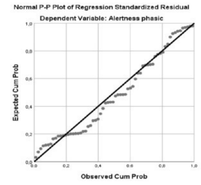
Normal P-P Plot of Regression Standardized Residual
Dependent Variable: Alertness phasic
Expected Cum Prob
Observed Cum Prob
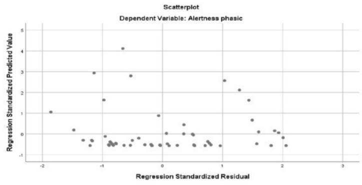
Scatterplot
Dependent Variable: Alertness phasic
Regression Standardized Predicted Value
Regression Standardized Residual
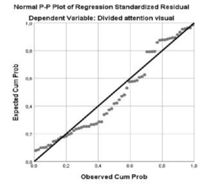
Normal P-P Plot of Regression Standardized Residual
Dependent Variable: Divided attention visual
Expected Cum Prob
Observed Cum Prob
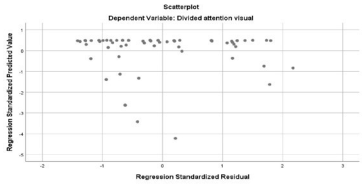
Scatterplot
Dependent Variable: Divided attention visual
Regression Standardized Predicted Value
Regression Standardized Residual
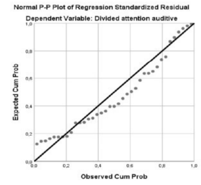
Normal P-P Plot of Regression Standardized Residual
Dependent Variable: Divided attention auditive
Expected Cum Prob
Observed Cum Prob
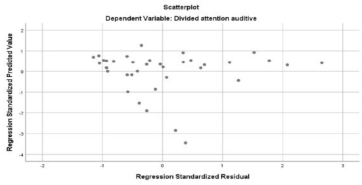
Scatterplot
Dependent Variable: Divided attention auditive
Regression Standardized Predicted Value
Regression Standardized Residual
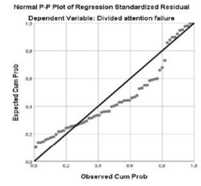
Normal P-P Plot of Regression Standardized Residual
Dependent Variable: Divided attention failure
Expected Cum Prob
Observed Cum Prob
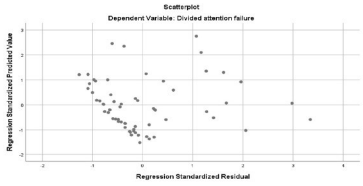
Scatterplot
Dependent Variable: Divided attention failure
Regression Standardized Predicted Value
Regression Standardized Residual
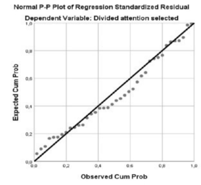
Normal P-P Plot of Regression Standardized Residual
Dependent Variable: Divided attention selected
Expected Cum Prob
Observed Cum Prob
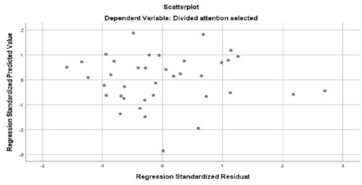
Scatterplot
Dependent Variable: Divided attention selected
Regression Standardized Predicted Value
Regression Standardized Residual
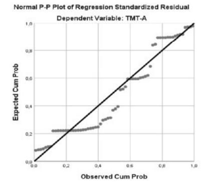
Normal P-P Plot of Regression Standardized Residual
Dependent Variable: TMT-A
Expected Cum Prob
Observed Cum Prob
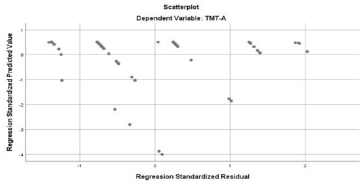
Scatterplot
Dependent Variable: TMT-A
Regression Standardized Predicted Value
Regression Standardized Residual
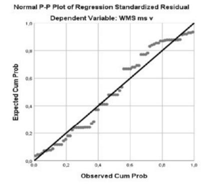
Normal P-P Plot of Regression Standardized Residual
Dependent Variable: WMS ms v
Expected Cum Prob
Observed Cum Prob
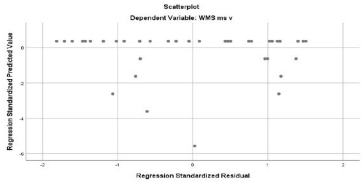
Scatterplot
Dependent Variable: WMS ms v
Regression Standardized Predicted Value
Regression Standardized Residual
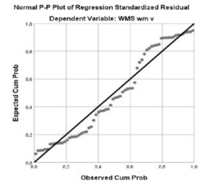
Normal P-P Plot of Regression Standardized Residual
Dependent Variable: WMS wm v
Expected Cum Prob
Observed Cum Prob
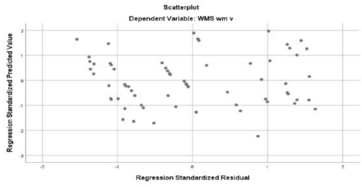
Scatterplot
Dependent Variable: WMS wm v
Regression Standardized Predicted Value
Regression Standardized Residual
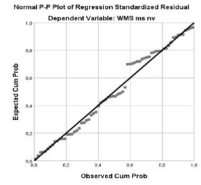
Normal P-P Plot of Regression Standardized Residual
Dependent Variable: WMS ms nv
Expected Cum Prob
Observed Cum Prob
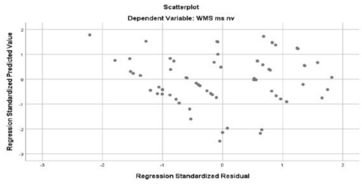
Scatterplot
Dependent Variable: WMS ms nv
Regression Standardized Predicted Value
Regression Standardized Residual
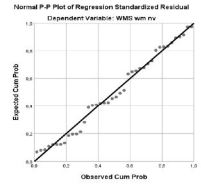
Normal P-P Plot of Regression Standardized Residual
Dependent Variable: WMS wm nv
Expected Cum Prob
Observed Cum Prob
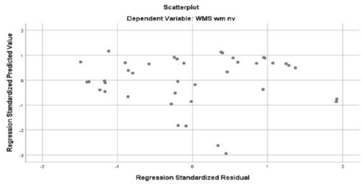
Scatterplot
Dependent Variable: WMS wm nv
Regression Standardized Predicted Value
Regression Standardized Residual

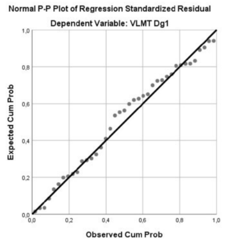
Normal P-P Plot of Regression Standardized Residual
Dependent Variable: VLMT Dg1
Expected Cum Prob
Observed Cum Prob

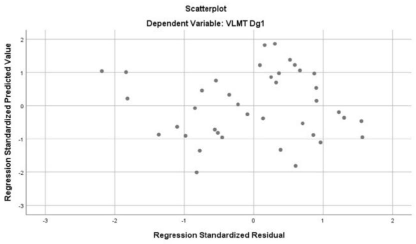
Scatterplot
Dependent Variable: VLMT Dg1
Regression Standardized Predicted Value
Regression Standardized Residual

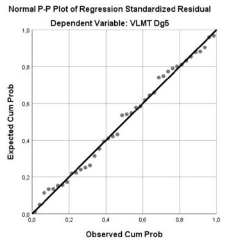
Normal P-P Plot of Regression Standardized Residual
Dependent Variable: VLMT Dg5
Expected Cum Prob
Observed Cum Prob

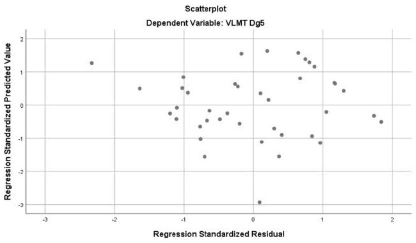
Scatterplot
Dependent Variable: VLMT Dg5
Regression Standardized Predicted Value
Regression Standardized Residual

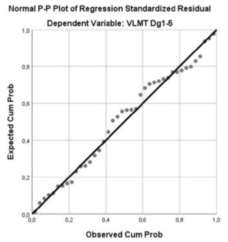
Normal P-P Plot of Regression Standardized Residual
Dependent Variable: VLMT Dg1-5
Expected Cum Prob
Observed Cum Prob

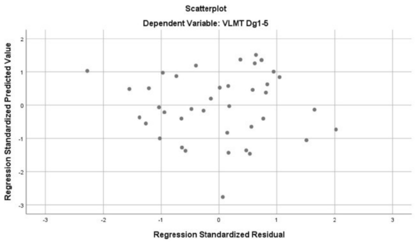
Scatterplot
Dependent Variable: VLMT Dg1-5
Regression Standardized Predicted Value
Regression Standardized Residual

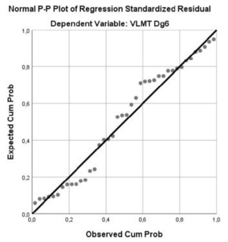
Normal P-P Plot of Regression Standardized Residual
Dependent Variable: VLMT Dg6
Expected Cum Prob
Observed Cum Prob

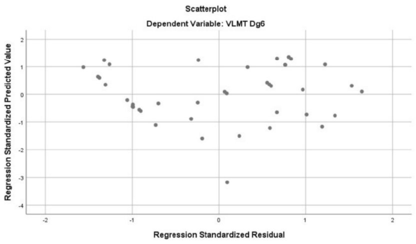
Scatterplot
Dependent Variable: VLMT Dg6
Regression Standardized Predicted Value
Regression Standardized Residual

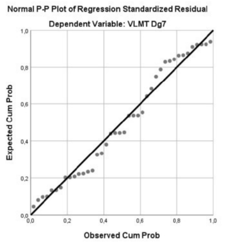
Normal P-P Plot of Regression Standardized Residual
Dependent Variable: VLMT Dg7
Expected Cum Prob
Observed Cum Prob

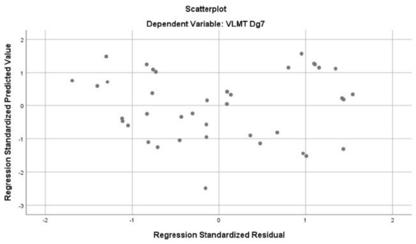
Scatterplot
Dependent Variable: VLMT Dg7
Regression Standardized Predicted Value
Regression Standardized Residual

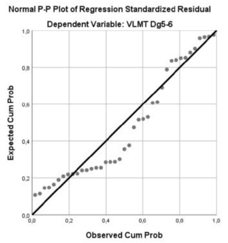
Normal P-P Plot of Regression Standardized Residual
Dependent Variable: VLMT Dg5-6
Expected Cum Prob
Observed Cum Prob

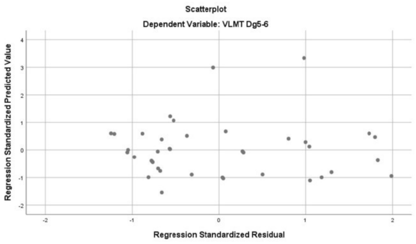
Scatterplot
Dependent Variable: VLMT Dg5-6
Regression Standardized Predicted Value
Regression Standardized Residual

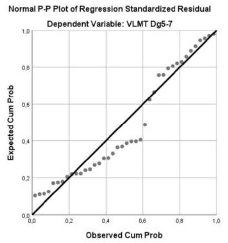
Normal P-P Plot of Regression Standardized Residual
Dependent Variable: VLMT Dg5-7
Expected Cum Prob
Observed Cum Prob

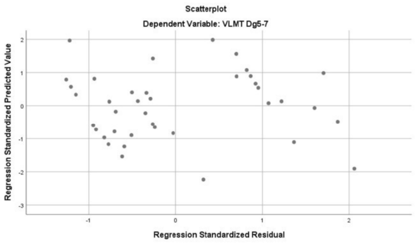
Scatterplot
Dependent Variable: VLMT Dg5-7
Regression Standardized Predicted Value
Regression Standardized Residual

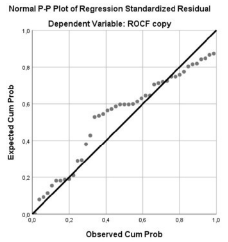
Normal P-P Plot of Regression Standardized Residual
Dependent Variable: ROCF copy
Expected Cum Prob
Observed Cum Prob

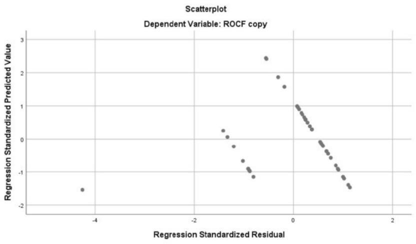
Scatterplot
Dependent Variable: ROCF copy
Regression Standardized Predicted Value
Regression Standardized Residual

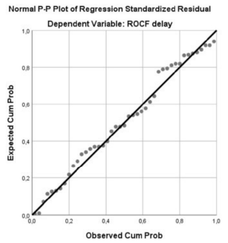
Normal P-P Plot of Regression Standardized Residual
Dependent Variable: ROCF delay
Expected Cum Prob
Observed Cum Prob

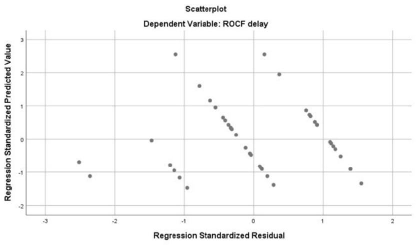
Scatterplot
Dependent Variable: ROCF delay
Regression Standardized Predicted Value
Regression Standardized Residual

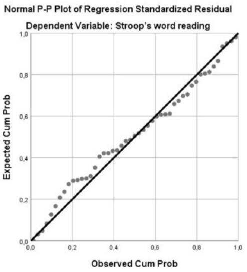
Normal P-P Plot of Regression Standardized Residual
Dependent Variable: Stroop's word reading
Expected Cum Prob
Observed Cum Prob

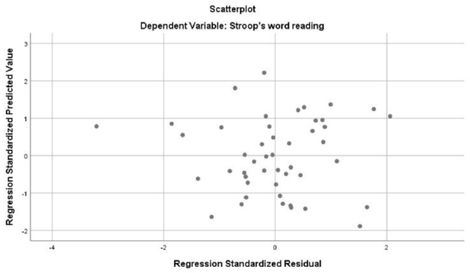
Scatterplot
Dependent Variable: Stroop's word reading
Regression Standardized Predicted Value
Regression Standardized Residual

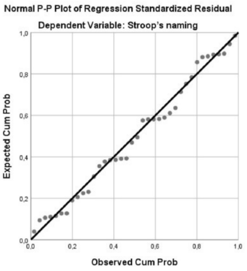
Normal P-P Plot of Regression Standardized Residual
Dependent Variable: Stroop's naming
Expected Cum Prob
Observed Cum Prob

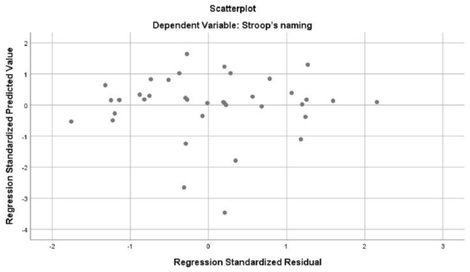
Scatterplot
Dependent Variable: Stroop's naming
Regression Standardized Predicted Value
Regression Standardized Residual

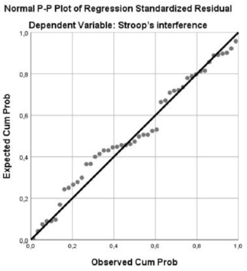
Normal P-P Plot of Regression Standardized Residual
Dependent Variable: Stroop's interference
Expected Cum Prob
Observed Cum Prob

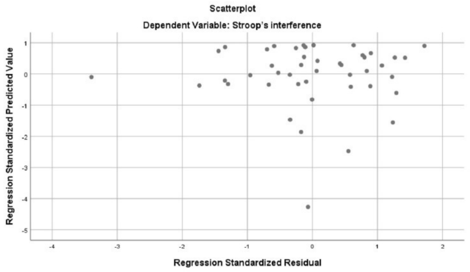
Scatterplot
Dependent Variable: Stroop's interference
Regression Standardized Predicted Value
Regression Standardized Residual

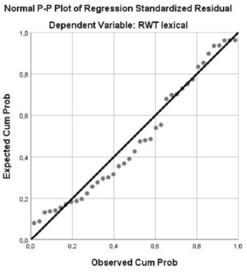
Normal P-P Plot of Regression Standardized Residual
Dependent Variable: RWT lexical
Expected Cum Prob
Observed Cum Prob

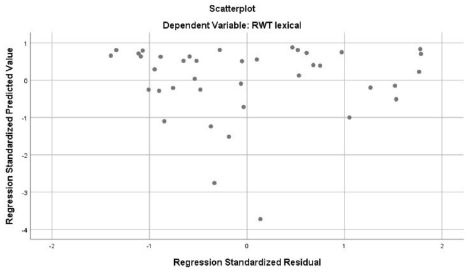
Scatterplot
Dependent Variable: RWT lexical
Regression Standardized Predicted Value
Regression Standardized Residual

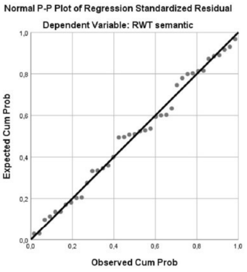
Normal P-P Plot of Regression Standardized Residual
Dependent Variable: RWT semantic
Expected Cum Prob
Observed Cum Prob

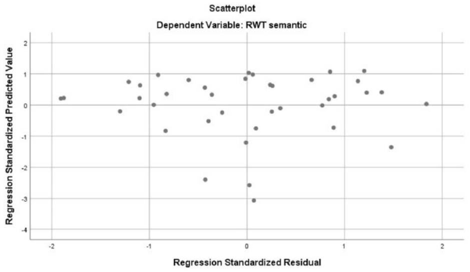
Scatterplot
Dependent Variable: RWT semantic
Regression Standardized Predicted Value
Regression Standardized Residual

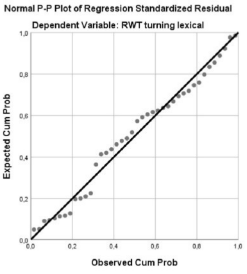
Normal P-P Plot of Regression Standardized Residual
Dependent Variable: RWT turning lexical
Expected Cum Prob
Observed Cum Prob

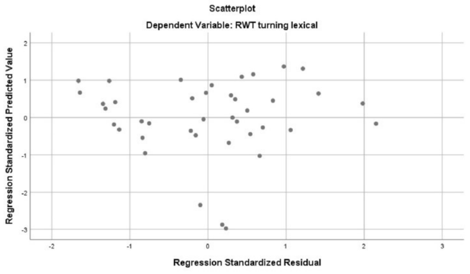
Scatterplot
Dependent Variable: RWT turning lexical
Regression Standardized Predicted Value
Regression Standardized Residual

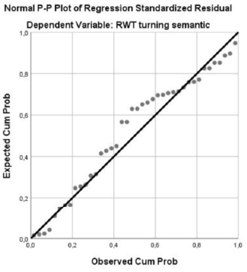
Normal P-P Plot of Regression Standardized Residual
Dependent Variable: RWT turning semantic
Expected Cum Prob
Observed Cum Prob

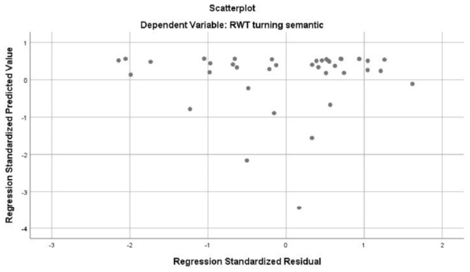
Scatterplot
Dependent Variable: RWT turning semantic
Regression Standardized Predicted Value
Regression Standardized Residual

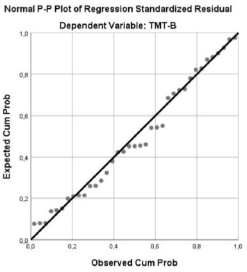
Normal P-P Plot of Regression Standardized Residual
Dependent Variable: TMT-B
Expected Cum Prob
Observed Cum Prob

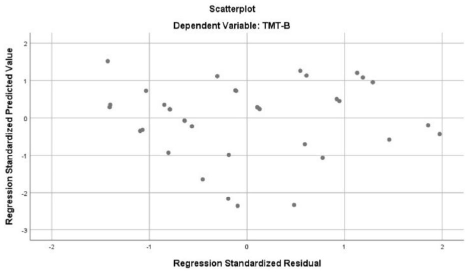
Scatterplot
Dependent Variable: TMT-B
Regression Standardized Predicted Value
Regression Standardized Residual
